# Feasibility and clinical applicability of genomic profiling based on cervical smear samples in patients with endometrial cancer

**DOI:** 10.3389/fonc.2022.942735

**Published:** 2022-08-05

**Authors:** Namsoo Kim, Yoo-Na Kim, Kyunglim Lee, Eunhyang Park, Yong Jae Lee, So Yoon Hwang, Jihyang Park, Zisun Choi, Sang Wun Kim, Sunghoon Kim, Jong Rak Choi, Seung-Tae Lee, Jung-Yun Lee

**Affiliations:** ^1^ Department of Laboratory Medicine, Yonsei University College of Medicine, Seoul, South Korea; ^2^ Department of Obstetrics and Gynecology, Institute of Women’s Life Medical Science, Yonsei University College of Medicine, Seoul, South Korea; ^3^ Department of Pathology, Yonsei University College of Medicine, Seoul, South Korea; ^4^ Dxome co., Ltd., Seongnam, South Korea

**Keywords:** endometrial cancer, Papanicolaou (PAP) smear, circulating tumor DNA (ctDNA), molecular classification and biomarkers, immunohistochemistry

## Abstract

**Purpose:**

Cervical smear samples are easily obtainable and may effectively reflect the tumor microenvironment in gynecological cancers. Therefore, we investigated the feasibility of genomic profiling based on tumor DNA analysis from cervical smear samples from endometrial cancer patients.

**Materials and methods:**

Preoperative cervical smear samples were obtained *via* vaginal sampling in 50 patients, including 39 with endometrial cancer and 11 with benign uterine disease. Matched blood samples were obtained simultaneously. Genomic DNA (gDNA) from cervical smear and/or cell-free DNA from whole blood were extracted and sequenced using the Pan100 panel covering 100 endometrial cancer-related genes.

**Results:**

Cervical swab-based gDNA analysis detected cancer with 67% sensitivity and 100% specificity, showing a superior performance compared to that of the matched blood or Pap smear tests. Cervical swab-based gDNA effectively identified patients with loss of MSH2 or MSH6 and aberrant p53 expression based on immunohistochemistry. Genomic landscape analysis of cervical swab-based gDNA identified *PTEN*, *PIK3CA*, *TP53*, and *ARID1A* as the most frequently altered genes. Furthermore, 26 endometrial cancer patients could be classified according to the Proactive Molecular Risk Classifier for Endometrial Cancer.

**Conclusion:**

Cervical swab-based gDNA test showed an improved detection potential and allowed the classification of patients, which has both predictive and prognostic implications.

## Introduction

Endometrial cancer originates in cells that form the inner lining of the uterus. Despite being the most common gynecological cancer worldwide, contributing to 12,550 deaths in the US in 2022, no effective screening tool for endometrial cancer has yet been available (1). While most patients who present with early-stage disease with low-grade endometrioid histology have a good prognosis, a subset of high-risk patients perform poorly, and is traditionally identified by histopathological analysis of post-hysterectomy specimens (2). Heralded by the Proactive Molecular Risk Classifier for Endometrial Cancer (ProMisE) (3), more efforts are being put forward to understand the genomic and proteomic alterations (4, 5) to help in early detection and risk stratification of patients with endometrial cancer (6). From a therapeutic perspective, the ability to precisely predict the prognosis (i.e., the ProMisE) prior to hysterectomy may aid in planning for surgery and adjuvant therapy.

From a diagnostic perspective, several non-invasive tools have been considered for endometrial cancer screening, including the qualitative assessment of the endometrium through a transvaginal ultrasound; measurement of blood-based serum markers, such as cancer antigen 125 (CA125); and Pap smear (7–9). However, to date, the diagnosis of endometrial carcinoma or its precursors relies on the pathologic evaluation of the endometrium based on diagnostic curettage. Endometrial biopsy is an invasive procedure that frequently requires general anaesthesia. Moreover, an adequate amount of sample is difficult to obtain, specifically in postmenopausal women in whom poor prognostic, non-hormone dependent endometrial cancer is relatively common (10, 11). Various imaging modalities, such as computed tomography (CT), magnetic resonance imaging, and positron emission tomography-CT have been used for preoperative assessment and postoperative monitoring (12). However, these imaging modalities suffer from drawbacks, such as high cost and exposure to harmful radiation, and have inherently limited scope as early detection tools because of their low sensitivity for microscopic tumors (13). To address these issues, a method for collecting circulating tumor DNA (ctDNA) from liquid biopsies has been developed (14, 15). ctDNA monitoring from the blood has helped in detecting cancer recurrence well in advance of pathological manifestations or imaging (16–18). However, whole blood ctDNA analysis could detect mutations based on plasma in only 18% of early-stage endometrial cancer patients, therefore indicating a limited capacity of blood-based tests as an early detection tool (19).

As an alternative liquid biopsy tool, previous proof-of-concept studies explored biological materials from endometrial cancer cells that shed through the cervix to diagnose and comprehend the tumor molecular architectures from the upper genital tract (20–22). This cytology brush-guided approach can be implemented by practice nurses in community healthcare settings and patients themselves and has the potential to be seamlessly integrated into an already existing cervical cancer screening program. Therefore, our objective was to utilise a comprehensive panel of 100 endometrial cancer genes based on cervical swab samples and ctDNA from matched whole blood samples to investigate the feasibility and clinical applicability of cervical swab-based genomic DNA (gDNA) analysis. We incorporated detailed clinical information, including immunohistochemistry (IHC)-based biomarkers, to evaluate the clinical relevance of the findings at genome levels.

## Materials and methods

### Patient recruitment

A total of 50 patients diagnosed with endometrial disease, including 39 patients with predominantly early-stage cancer (20 stage IA patients and 11 stage III or IV patients) and 11 patients without cancer (five with endometrial hyperplasia and six with benign uterine disease), were prospectively enrolled. Specifically, women diagnosed with endometrial carcinoma who underwent staging operation at Severance Hospital from January 2021 to October 2021 were recruited. Patients with any other gynecological malignancies such as ovarian or cervical cancer were excluded. From the same time window, women undergoing resectoscope-guided procedures for suspected premalignant endometrial disease and age-matched women with benign endometrial pathology were also included. Conventional Pap smear results were obtained before operation and reviewed for the presence of carcinoma. Cervical swab-based gDNA samples were obtained in the operating room using a Cervex-Brush (Rovers Medical Device, Noord-Brabant, Netherlands). After anaesthesia, a blind swab of the vagina was performed without using a speculum. Simultaneously, 10 cc of matched whole blood sample was drawn from each patient. This study was conducted according to the guidelines of the Declaration of Helsinki and was approved by the Institutional Review Board of the Severance Hospital, Seoul, Korea (approval no. 4-2020-1265).

### Acquisition of cell-free DNA and next-generation sequencing

For tumor gDNA in cervical swab analysis, we used a brush inside a 50 ml conical tube with saline. The cervical swab was centrifuged for 10 min at 1,900 × g, and the pellet was resuspended, leaving only 1 mL of the supernatant. For blood ctDNA analysis, we collected 10 ml of blood using the Dxtube (Dxome Co. Ltd., Seongnam, Gyeonggi-do, Republic of Korea) and DNA was extracted within 4 days. The blood sample was centrifuged for 15 min at 1,900 × g. Then, the supernatant was centrifuged again for 10 min at 1,900 × g, and the resulting supernatant was collected and stored at -80°C. Cell-free DNA was extracted from plasma using Magnetic Circulating DNA Maxi Reagent (Dxome). Genomic DNA was extracted using QIAamp DNA Mini Kit (51306, QIAGEN, Hilden, Germany) from 200 uL buffy coat derived from whole blood or 200 uL cervical swab-cell suspension. Final elution was performed in 100 uL, and 1000 ng of gDNA was sheared to 200–250 base pairs of average size. Size and quantitative measurements were performed using the D1000 ScreenTape system (Agilent, CA, USA). Library was prepared using the DxLiquid NGS system for Illumina (Cat No. LP01096, Dxome). We used 5–30 ng ctDNA for library preparation, and the amplification cycle depended on the amount of input ctDNA. A target enrichment library was generated using the DxLiquid Pan100 (Cat no. DL-AO1001024, Dxome) according to the manufacturer’s instructions. Library sequencing was performed using Illumina instruments (Novaseq or Nextseq2000) (Illumina, CA, USA).

Sequencing data were analysed using the position indexing sequencing (PiSeq) algorithm, which adopts genome position of the sequencing reads and refine the accuracy of molecular barcoding to enable accurate determination of variants with low variant allele frequency (VAF). Variants were classified into four tiers based on the Association for Molecular Pathology with liaison representation from the American College of Medical Genetics and Genomics, the American Society of Clinical Oncology, and the College of American Pathologists (23). Classifying variants included the web databases OncoKB, cBioPortal, and My Cancer Genome (24–26). All tier I and II variants were visually confirmed using Integrated Genomics Viewer.

### Collection of clinical variables and immunohistochemistry profile

Endometrium samples from operations, hysterectomy, resectoscope, or dilatation and curettage, were assessed by an expert pathologist. Among 50 patients contributing tissue specimen, 29 patients also underwent IHC profiling using formalin-fixed, paraffin-embedded tissue specimens. After deparaffinization with xylene and rehydration with graded alcohol solution, IHC was performed using Ventana Discovery XT Automated Slide Stainer (Ventana Medical System, Tucson, AZ, USA). Cell Conditioning Buffer 1 (citrate buffer, pH 6.0; Ventana Medical System) was used for antigen retrieval. The slices were incubated with primary antibodies against MLH1 (dilution 1:50, BD Biosciences, San Jose, CA, USA), MSH2 (dilution 1:200, BD Biosciences), MSH6 (dilution 1:100, Cell Marque Corporation, Rocklin, CA, USA), PMS2 (dilution 1:40, Cell Marque), and p53 (dilution 1:50, Dako, CA, USA). IHC stain was scored and interpreted by an expert pathologist (E Park).

### Statistical analysis

Statistical analysis was performed using R. Statistical significance was calculated using Fischer’s exact test or chi-squared test for categorical variables and Student’s *t*-test for continuous variables. The McNemar test with continuity correction was used for the analysis of matched samples. The genomic data in a form of variant calling file from the afore-mentioned pipeline were analysed using the R package “maftools” (27).

## Results

**Figure 1:**
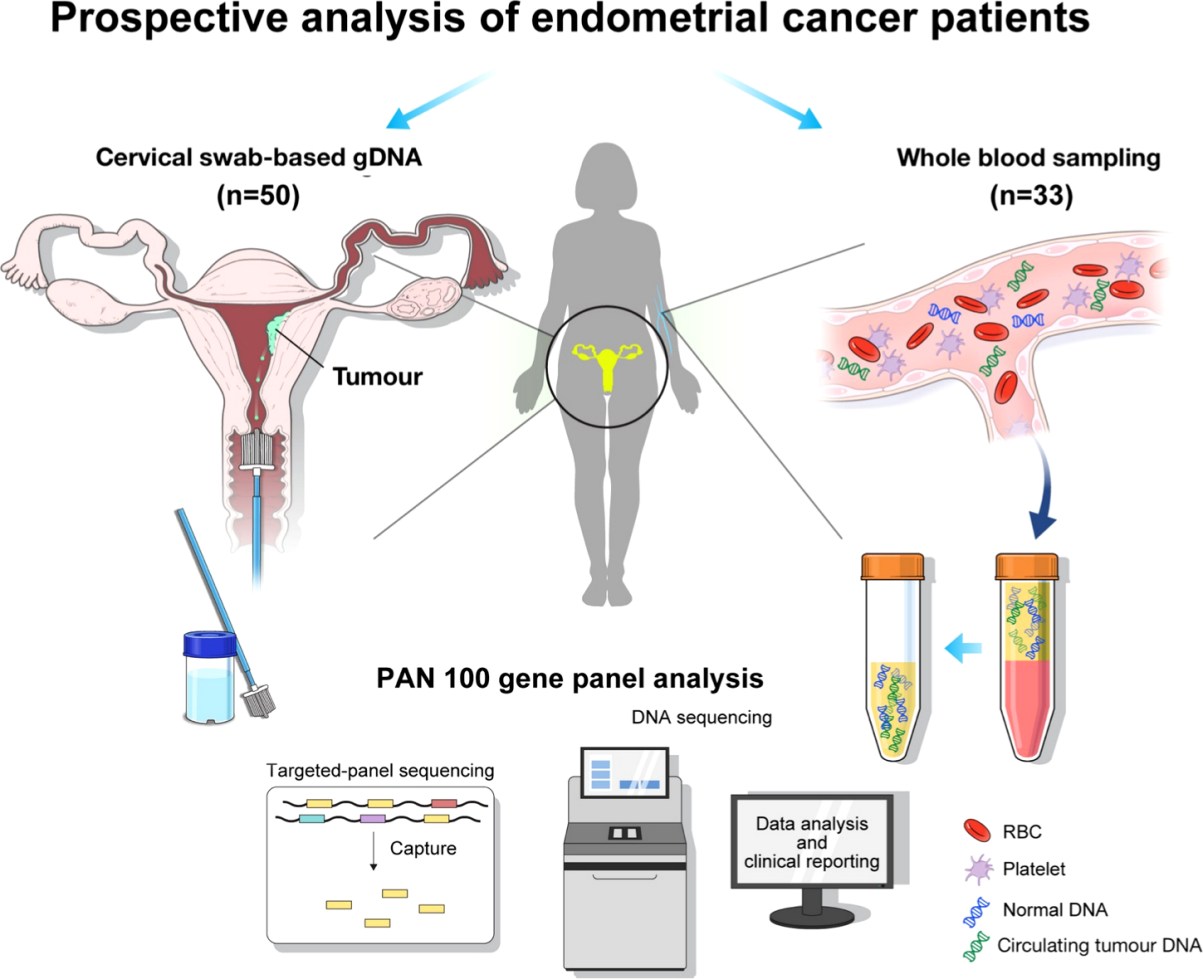
Graphical summary of the analysis flow: the collection of genomic DNA (gDNA) based on cervical swab and circulating tumor DNA (ctDNA) based on whole blood sampling, followed by genomic profiling with Pan100 panel.

We obtained 50 cervical swab samples from as many patients for gDNA analysis and 33 matched blood samples for sequencing (**Figure 1**). Of the 50 patients with cervical swab samples, 39 had endometrial cancer and 11 had benign diseases, including endometrial hyperplasia and benign endometrial pathology (**Table 1**). Among 33 patients with matched blood samples, 31 had endometrial cancer and 2 had benign disease. Based on the final pathology of 50 patients, one-fourth of patients were classified as low risk (i.e., satisfying stage IA disease, low-risk histology, and tumor size of less than 2 cm). Conversely, disease outside the uterus was found in 28.2% of patients; non-endometrioid high-risk histology was identified in five patients, including four with sarcoma and one with serous histology. Analysis of cervical swab-based gDNA correctly identified 26 of 39 endometrial cancer patients, with 67% sensitivity; no false-positive cases were identified among 11 patients with benign pathology, corresponding to 100% specificity (**Table 2**). Among 13 cases that were missed by cervical swab-based gDNA, 11 patients had stage IA disease. Excluding patients with stage IA cancer, the sensitivity was 89% (**Supplementary Table 1**).

**Table 1:** Distribution of clinical variables among patients with endometrial cancer.

Variables	All patients (n=50)
Age, year (mean ± SD)	55.7 ± 12.2
Clinical Stage (n, %)	
IA IB II III IV AH/EIN Benign	20 (40%) 7 (14%) 1 (2%) 7 (14%) 4 (8%) 5 (10%) 6 (12%)
	**Endometrial cancer patients (n=39)**
Histology, n (%)	
Endometrioid G1 Endometrioid G2 Endometrioid G3 Serous Sarcoma	10 (26%) 20 (51%) 5 (13%) 1 (3%) 3 (8%)
Tumour size, cm (median, range)	2.5 (0–14)
Myometrial invasion depth	
Minimal Less than half More than half	9 (23%) 12 (31%) 18 (46%)
Lymph node metastasis	
No Yes	43 (86%) 7 (14%)
Immunohistochemistry, n (%)	
MMRd (loss of protein expression) PTEN loss p53 aberrant expression	9 (out of 29, 31%) 6 (out of 11, 55%) 5 (out of 22, 23%)

SD standard deviation, MMRd mismatch repair deficiency.

**Table 2:** Performance of cervical swab-based genomic DNA (gDNA) for predicting endometrial cancer versus benign uterine disease.

		Reference	
		No cancer	Cancer
Prediction by cervical swab-based gDNA	Negative	11	13
	Positive	0	26
Accuracy: 0.74 (95% CI 0.60 – 0.85)			
Sensitivity: 0.67			
Specificity: 1.00			
Positive predictive value: 1.00			
Negative predictive value: 0.46			

Overall, among 39 patients of endometrial cancer, cervical-swab-based gDNA test successfully deleted genomic alterations in 26 patients. Comparison of clinical factors among detected and undetected patients showed that patients with abnormal findings on cervical swab-based gDNA had a relatively large mass or deep myometrial invasion (**Table 3**). Furthermore, cervical swab-based gDNA assessment showed an overall accuracy of 95% (95% CI of 0.83–0.99), with a sensitivity of 93% and specificity of 100% (**Table 4**) with respect to cervical swab or whole blood ctDNA as a reference. Among the 13 patients not detected by cervical-swab-based gDNA, two had sarcoma; all 13 patients underwent Pap smear test and seven had matched blood test. Among these false-negative cases, one patient was identified as having carcinoma based on a Pap smear, and one was detected with carcinosarcoma histology based on whole blood ctDNA with negative Pap smear results. Detailed clinical profiles of the patients are shown in **Supplementary Table 2**.

**Table 3:** Comparison of clinicopathological factors between patients who are detected verses those who are not detected by cervical swab-based genomic DNA (gDNA) among patients with endometrial cancer.

	Cervical swab-based gDNA detection		
	Negative (n=13)	Positive (n=26)	*p*-value
Age, year (mean ± SD)	57.9 ± 10.3	57.4 ± 11.7	0.8844
Histology risk			0.4472
Low (Endometrioid G1) High (Endometrioid G2/3 or non-endometrioid histology type)	9 (69%) 4 (31%)	21 (81%) 5 (19%)	
Tumour size, cm (median, range)	1.5 (0–3.8)	3.3 (0.4–14)	<0.0001
Myometrial invasion			0.0025
Minimal Less than half More than half	7 (54%) 4 (31%) 2 (15%)	2 (8%) 8 (31%) 16 (62%)	
Clinical stage			0.03203
IA IB II III IV	11 (84%) 0 (0%) 0 (0%) 1 (8%) 1(8%)	9 (35%) 7 (27%) 1 (4%) 6 (23%) 3 (12%)	

**Table 4:** Performance of cervical swab-based genomic DNA (gDNA) for detection among endometrial cancer patients. .

		Reference*	
		Not detect by any test modality	Detect by any test modality
Prediction by cervical swab-based gDNA	Negative	11	2
	Positive	0	26
Accuracy: 0.95 (95% CI 0.8268 – 0.9937)			
Sensitivity: 0.93			
Specificity: 1.00			
Positive predictive value: 1.00			
Negative predictive value: 0.85			

*Reference based on detection by any of the two modalities (cervical swab-based gDNA or whole blood-based circulating tumor DNA).

**Figure 2:**
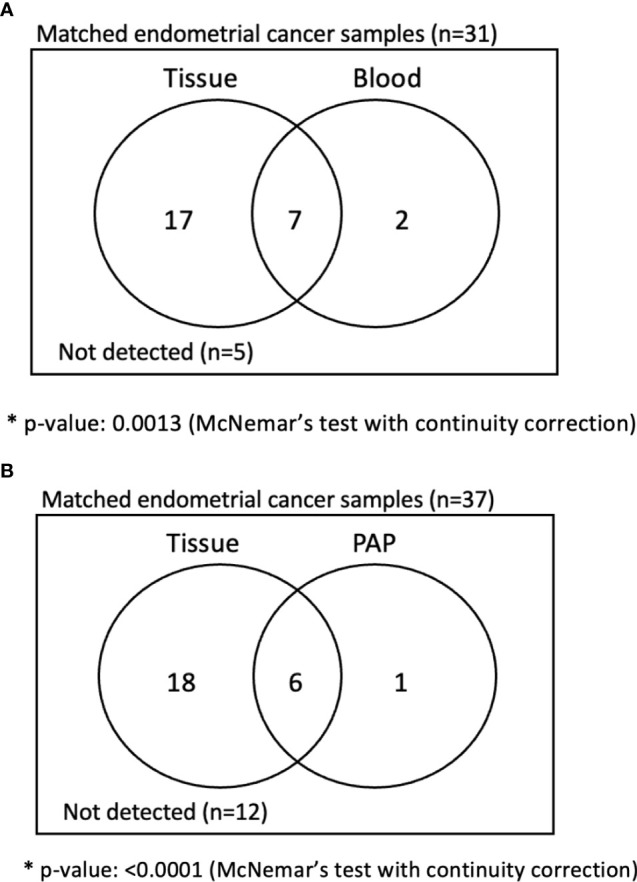
Analysis of matched sample in endometrial cancer patients. **(A)** Comparison between cervical swab-based genomic DNA (gDNA) versus whole blood-based circulating tumor DNA (ctDNA). **(B)** Comparison between cervical swab-based gDNA versus conventional Pap smear.

We then directly compared the detection rates of cervical swab-based gDNA and whole blood ctDNA tests among the matched samples (**Figure 2A**). Among the 31 patients who underwent both tests, cervical swab-based gDNA test identified 24 patients, and 17 were missed by whole blood test. Conversely, among seven patients who had negative findings from cervical swab-based gDNA test, whole blood test identified abnormalities in two patients. Cervical swab-based gDNA test showed a higher detection rate than the whole blood ctDNA test (69.2% vs. 30.8%, *p* value of 0.001 based on McNemar test) or Pap smear (66.7% vs. 17.9%, *p* value <0.0001 based on McNemar test). Similarly, cervical swab-based gDNA test performed better than conventional Pap smear test (**Figure 2B**).

IHC identified a loss of expression of any one of the four MMRd-associated proteins in eight out of 26 patients (30.8%): MSH2 or MSH6 mutations were found in five and PMS2 mutations were noticed in three additional patients. Genomic analysis based on cervical swab-based gDNA detected MSH2 or MSH6 mutation in four out of five patients. A representative IHC picture from a patient with MSH6 loss of expression and MSH6 mutation based on cervical swab-based gDNA (EMC_051) is shown in **Supplementary Figure 1**. Unlike MSH2 or MSH6, PMS2 mutations were not detected despite the coverage based on the Pan100 panel. All four patients with germline MMRd mutations from whole-blood-based ctDNA were identified using cervical swab-based gDNA. IHC identified aberrant p53 expression in four out of 19 patients (21.1%); three of them were found to have *TP53* mutations also based on cervical swab-based gDNA test. Among the three patients whose p53 mutation was identified by cervical swab-based gDNA test, two were not detected by matched whole blood ctDNA test. Moreover, cervical swab-based gDNA helped in identifying five additional patients with p53 mutations who were not detected by either IHC or whole blood-based ctDNA test.

**Figure 3:**
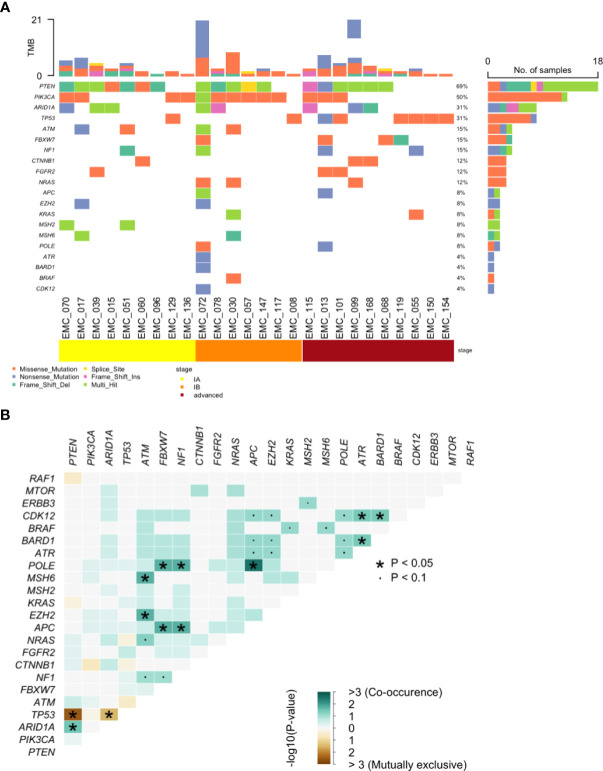
Genomic analysis of pathogenic mutations. **(A)** Landscape of somatic alterations and **(B)** Somatic interaction plot based on cervical swab-based gDNA from 26 patients.

**Figure 4:**
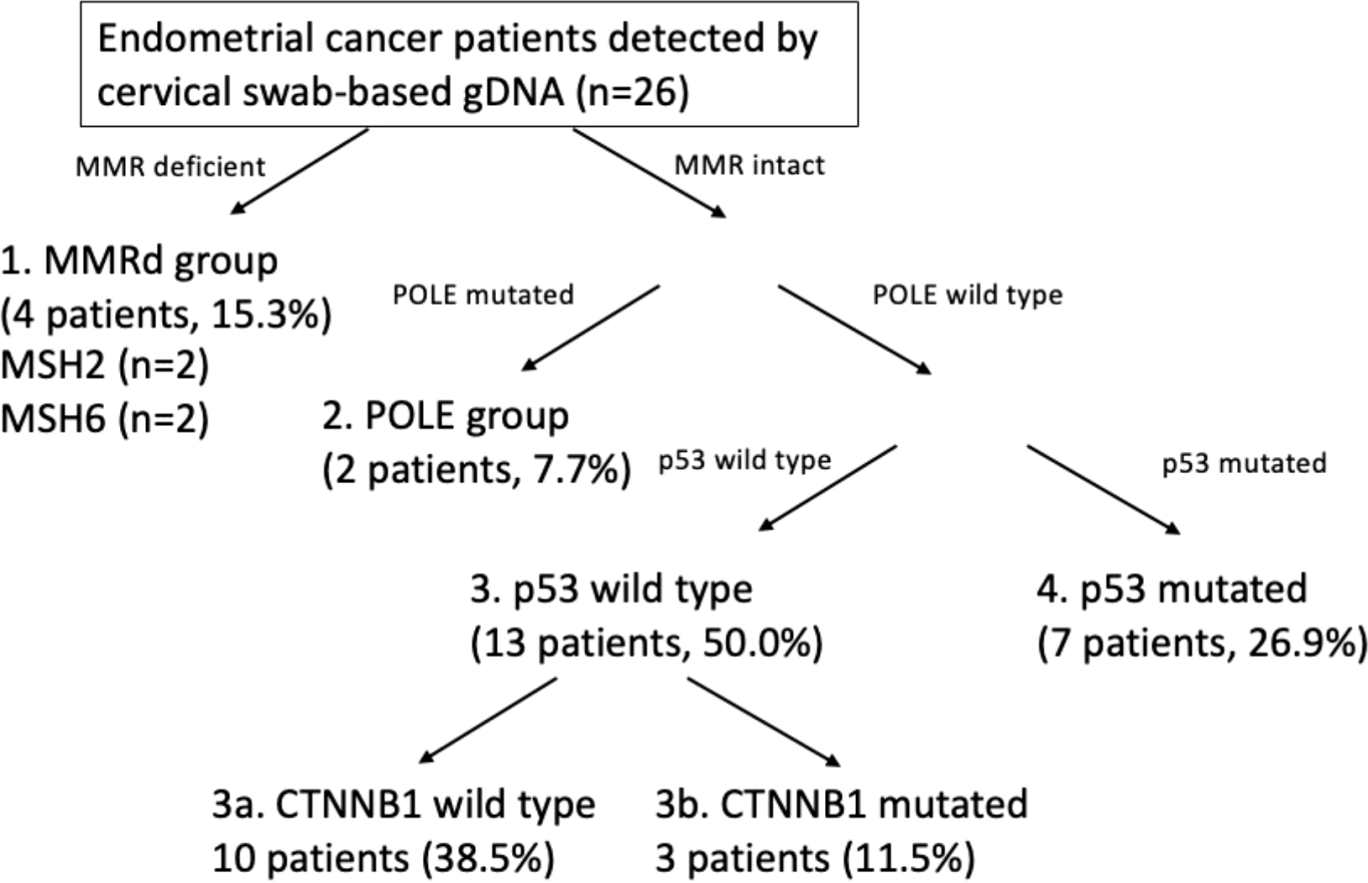
Application of the Proactive Molecular Risk Classifier for Endometrial Cancer (ProMisE) based on cervical swab-based genomic DNA.

The overall genomic mutation landscape, stratified based on the test modality and clinical stage, is shown in **Figure 3A**. The most frequently altered genes were *PTEN* (69%), *PIK3CA* (50%), *ARID1A* (31%), and *TP53* (31%). Although not frequent, genes with clinical implications included *CTNNB1* (n=3), *POLE* (n=2), *MSH2* (n=2), and *MSH6* (n=2), which were captured exclusively by cervical swab-based gDNA test. The most frequently altered genes per test modality (cervical swab-based gDNA vs. whole-blood-based ctDNA) are shown in **Supplementary Figure 1A**. Irrespective of the test modality, the most frequently highlighted pathways were the PI3K, TP53, and RTK-RAS pathway, in decreasing order of frequency (**Supplementary Figure 1B**). The specific location of each gene mutation is shown in **Supplementary Figure 2**. The somatic interaction plot highlighted co-occurring genes, such as *ARID1A* and *PTEN*, and mutually exclusive genes, such as *TP53* and *PTEN* (**Figure 3B**). Based on the genomic landscape and somatic interaction plot, a total of 26 patients with positive cervical swab-based gDNA test were classified based on gDNA mutation according to the ProMisE (**Figure 4**). These patients were subdivided into the MMRd (four patients, 15.3%), POLE (two patients, 7.7%), p53 abnormal (seven patients, 26.9%), and p53 wild-type (13 patients, 50.0%). Furthermore, the *CTNNB1* mutation, which was exclusively found in p53 wild-type patients, could be used to identify patients with relatively poor prognosis (n=3) among the p53 wild-type subgroup.

## Discussion

Our results show that genomic profiling of endometrial cancer patients based on cervical swabs is feasible and its performance is better than that of matched samples, either whole blood-based ctDNA or conventional Pap smears. Moreover, utilising a comprehensive panel of 100 endometrial-related genes, we found that *MMRd* genes (*MSH2* and *MSH6*) and *p53* cervical swab-based gDNA mutations were concordant with the IHC results. Genomic information based on cervical swab-based gDNA could be utilised to classify patients into four groups according to the ProMisE. Since we prospectively enrolled all patients with endometrial cancer, our result is reflective of real-world evidence of predominantly stage IA, low-risk patients visiting a tertiary centre. Sample collection using a simple vaginal swab further highlights that our approach can be introduced in actual clinical settings with minimal discomfort to the patients compared to sampling for endometrial biopsy and whole blood sampling which are invasive.

Previous proof-of-concept studies for non-invasive sampling of endometrial cancer patients utilised uterine lavage (21, 22) or cervical smear samples from Pap or Tao brush (28). In a study by Nair et al., based on the analysis of uterine lavage fluid with a panel of 12 genes, the sensitivity was 100% (7/7) and the specificity was 46% (44/95). Wang et al., through the analysis of cervical smear with a Tao brush with a panel of 18 genes, found that the sensitivity was 93% (114/123) and specificity was 100% (125/125). Another study by Wang et al. showed that with a Pap brush, the sensitivity was 81%, which was slightly lower than that of a Tao brush. Compared with these studies, our study showed a moderate sensitivity of 66.7% and a high specificity of 100%. Nonetheless, our pilot cohort predominantly consisted of patients with early-stage disease. Since the cervical swab-based gDNA detection rate is significantly high in patients with large tumor size and deep myometrial invasion, excluding stage IA patients would have led to a relatively high performance.

Moreover, we only included Tier 1 and Tier 2 genes with a conservative cut-off for VAF, to only capture the pathogenic somatic mutations for analysis. Cancer-associated mutations can be found in the normal human endometrium (29) and ultra-deep sequencing may help in identifying women with carrier mutations without clinically defined cancer diagnosis (21) including Tier 3, which may have improved our sensitivity or helped in detecting mutations in non-cancerous diseases. However, in this pilot study, we adopted a conservative approach. From a technical standpoint, our sensitivity can be improved with the assistance of a speculum device or the choice of brushes.

It is interesting to note that the cervical swab-based gDNA significantly outperformed matched whole blood ctDNA despite the use of the same gene panel and VAF cut-off. Moreover, the most frequently mutated genes differed between the two testing modalities. One possibility is that cervical swab-based gDNA is able to directly capture the spillage of tumor from endometrium. The relative advantage of anatomical proximity may be more pronounced in case of early-stage tumor because superficial tumor of the endometrium may not have spread to the blood stream. In addition to the depth of invasion, the tumor biology may have an effect; non-endometrioid endometrial cancer such as sarcoma or serous type may behave differently from endometrioid type. For instance, tumor cells from sarcoma may have a predilection for spread through blood stream at a relative earlier stage, leading to a higher detection rate by whole blood ctDNA compared to cervical swab-based gDNA. We do not have enough sample number for non-endometrioid histology types to make a conclusive statement yet. Further study with a larger sample size, including sizable patients with non-endometrioid type, will help assess this observation.

Our study is unique in that our gene panel was comprehensive and specifically designed for endometrial cancer. For instance, *MMRd* genes (*MSH2, MSH6, PMS2*) were not included in the two afore-mentioned studies (21, 28) as well as in a recent study utilising a panel of 127 driver genes from TCGA (30). POLE was covered in the study by Wang et al. but not in the other two studies. Since we performed a pilot study, in addition to the mere detection rate, we wanted to check the frequency of genes that have predictive and prognostic implications for endometrial cancer patients and the concordance of these genes with the IHC profile. Our findings showed that the loss of PMS2 on IHC was not captured by cervical swab-based gDNA test, yet the concordance with IHC was high for MSH2, MSH6, and p53. Since MMR deficiency can be caused by genomic and non-genomic causes, such as MLH1 methylation, that may lead to the loss of expression evident in IHC, our gDNA analysis was not able to capture these non-genomic causes. Moreover, mutations with low copy number variation or VAF could have been missed by gDNA analysis. Therefore, we might not capture all MMRd cases, but we were still able to capture 15.3% of the patients in the MMRd group based solely on cervical swabs. Recent reports on the validation cohort of the ProMisE reported MMRd 28.1%, POLE 9.3%, p53 wild type 50.4%, and p53 abnormal 12.2% (3), which is comparable to our data (MMRd 15.3%, POLE 7.7%, p53 wild-type 50.0%, and p53 abnormal 26.9%). Moreover, cervical swab-based gDNA also detected *CTNNB1* mutations exclusively among p53 wild-type cases, enabling the identification of a poor prognostic subgroup among the p53 wild-type group. The ability to correctly identify the MMRd status or even categorise patients according to the ProMisE prior to surgical staging or hysterectomy provides a significant advantage to clinicians in terms of treatment planning. In addition to patients with advanced-stage endometrial cancers and needing adjuvant therapy, early-stage cancers patients who consider fertility sparing, the MMRd status may help tailor treatment such as the use of hormonal therapy or intrauterine devices (31).

Somatic interaction analysis showed that our data are consistent with those of previous reports. For instance, *PTEN* and *TP53* have been reported to be mutually exclusive in breast cancer stroma (32) and other cancer types; however, further studies are necessary in endometrial cancer (33). Moreover, *ARID1A* and *TP53* are mutually exclusive in endometrial cancer (34). Lastly, *ARID1A* mutations have also been reported to frequently harbour PTEN or *PIK3CA* mutations, which have been implicated in carcinogenesis (35). Based on these scientific grounds, among *TP53* wild-type patients, we used a combination of mutations in these three genes (*PTEN, ARID1A, PIK3CA*), which were also the most frequently identified genes. With a large sample size, the analysis of genomic information may provide insight into endometrial carcinogenesis. In clinical settings, genetic information captured on cervical swab-based gDNA has direct implications on precision therapy and prognosis stratification for patients with endometrial cancer.

The limitation of our study is that we included a relatively small sample size. Our cohort predominantly consisted of stage IA patients, yet this distribution is reflective of real-world data. Our method of blind sampling may have led to false-negative findings, and utilising speculum devices may improve the detection rate. Other brush types, such as the Tao brush, may be more useful for the direct collection of samples from the uterine cavity, although they incur potential discomfort to patients compared to the Cervex brush. Since this was a pilot study, validation with a large sample size is necessary.

Our pilot study shows that shredded cells from the uterine cavity can be captured by cervical swabs with sufficient tumor-derived genomic materials for comprehensive panel-based analysis. This cervical swab-based approach is less invasive and more accurate than the blood-based ctDNA approach. Cervical swab-based gDNA analysis can check for MMRd and p53 status, such that endometrial cancer patients can be categorised according to the ProMisE prior to undergoing hysterectomy, and therefore, has the potential to allow precision therapy for patients with endometrial cancer.

## Data availability statement

The original contributions presented in the study are included in the article/**Supplementary Material**. Further inquiries can be directed to the corresponding authors.

## Ethics statement

The studies involving human participants were reviewed and approved by Severance Hospital, Yonsei University, Seoul, South Korea. The patients/participants provided their written informed consent to participate in this study.

## Author contributions

Study was conceived and designed by JC, S-TL and J-YL. Pathological review of data was performed by EP. Clinical perspective was provided by KL, YJL, SWK and SK. Experiment was performed by SH, JP and ZC. Interpretation of the data was performed by NK, Y-NK, S-TL and JL. Analysis and interpretation of the data were performed by NK, Y-NK, S-TL and JL. Drafting of the manuscript was undertaken by NK and Y-NK. Critical review and revision of manuscript was undertaken by S-TL and JL. All authors contributed to the article and approved the submitted version.

## Funding

This study was supported by a Severance Hospital Research fund for clinical excellence (SHRC) (C-2022-0013).

## Acknowledgments

The authors would like to thank MID (Medical Illustration & Design), a part of the Medical Research Support Services of Yonsei University College of Medicine, for providing excellent support with medical illustration.

## Conflict of interest

SH, JP, ZC, JC, and S-TL are employees of Dxome co., Ltd.

The remaining authors declare that the research was conducted in the absence of any commercial or financial relationships that could be construed as a potential conflict of interest.

## Publisher’s note

All claims expressed in this article are solely those of the authors and do not necessarily represent those of their affiliated organizations, or those of the publisher, the editors and the reviewers. Any product that may be evaluated in this article, or claim that may be made by its manufacturer, is not guaranteed or endorsed by the publisher.

## Supplementary material

Supplementary Figure 1 Representative IHC picture from a patient with MSH6 loss of expression and MSH6 mutation based on cervical swab-based gDNA (EMC_051).

Supplementary Figure 2 Genetic alteration profile. **(A)** Most commonly altered genes based on cervical swab-based genomic DNA (left) and whole blood-based circulating tumor DNA (right). **(B)** Frequently highlighted pathways based on cervical swab-based gDNA from 26 patients. Number of patients with affected pathway is shown on the right; fraction of patients with affected pathway is shown on the bottom. **(C)** Top 3 most frequently mutated pathway, including specific genes involved.

Supplementary Figure 3 Lollipop plot of somatic nucleotide level mutations for genes, including *PTEN*, *PIK3CA, TP53, MSH2, MSH6, and POLE* based on cervical swab-based gDNA.

## References

[1] Siegel RL Miller KD Fuchs HE Jemal A. Cancer statistics, 2022. CA: A Cancer J Clin (2022) 72(1):7–33. doi: 10.3322/caac.21708 35020204

[2] Monk BJ Smith G Lima J Long GH Alam N Nakamura H. Real-world outcomes in patients with advanced endometrial cancer: A retrospective cohort study of US electronic health records. Gynecol Oncol (2022) 164(2):325–32. doi: 10.1016/j.ygyno.2021.12.008 34952707

[3] Kommoss S McConechy MK Kommoss F Leung S Bunz A Magrill J. Final validation of the ProMisE molecular classifier for endometrial carcinoma in a large population-based case series. Ann Oncol (2018) 29(5):1180–8. doi: 10.1093/annonc/mdy058 29432521

[4] Getz G Gabriel SB Cibulskis K Lander E Sivachenko A Sougnez C. Integrated genomic characterization of endometrial carcinoma. Nature (2013) 497(7447):67–73. doi: 10.1038/nature12113 23636398 PMC3704730

[5] Beinse G le Frere Belda MA Just PA Bekmezian N Koual M Garinet S. Development and validation of a RNAseq signature for prognostic stratification in endometrial cancer. Gynecol Oncol (2022) 164(3):596–606. doi: 10.1016/j.ygyno.2022.01.005 35033379

[6] Tanos P Dimitriou S Gullo G Tanos V. Biomolecular and genetic prognostic factors that can facilitate fertility-sparing treatment (FST) decision making in early stage endometrial cancer (ES-EC): A systematic review. Int J Mol Sci (2022) 23(5):2653. doi: 10.3390/ijms23052653 35269800 PMC8910305

[7] Wong M Amin T Thanatsis N Naftalin J Jurkovic D. A prospective comparison of the diagnostic accuracies of ultrasound and magnetic resonance imaging in preoperative staging of endometrial cancer. J Gynecol Oncol (2022) 33(2):e22. doi: 10.3802/jgo.2022.33.e22 35128854 PMC8899878

[8] Frias-Gomez J Tovar E Vidal A Murgui L Ibáñez R Peremiquel-Trillas P. Sensitivity of cervical cytology in endometrial cancer detection in a tertiary hospital in Spain. Cancer Med (2021) 10(19):6762–6. doi: 10.1002/cam4.4217 PMC8495290 34480514

[9] Reijnen C IntHout J Massuger LFAG Strobbe F Küsters-Vandevelde HVN Haldorsen IS. Diagnostic accuracy of clinical biomarkers for preoperative prediction of lymph node metastasis in endometrial carcinoma: A systematic review and meta-analysis. Oncol (2019) 24(9):e880–90. doi: 10.1634/theoncologist.2019-0117 PMC6738307 31186375

[10] Braun MM Grumbo RJ. Diagnosis and management of endometrial cancer. Am Fam Physician(2016) 93(6):468–74. 26977831

[11] Kim DH Seong SJ Kim MK Bae HS Kim M Yu BS. Dilatation and curettage is more accurate than endometrial aspiration biopsy in early-stage endometrial cancer patients treated with high dose oral progestin and levonorgestrel intrauterine system. J Gynecol Oncol (2017) 28(1):e1. doi: 10.3802/jgo.2017.28.e1 27670255 PMC5165062

[12] Ironi G Mapelli P Bergamini A Fallanca F Candotti G Gnasso C. Hybrid PET/MRI in staging endometrial cancer: Diagnostic and predictive value in a prospective cohort. Clin Nucl Med (2022) 47(3):E221–9. doi: 10.1097/RLU.0000000000004064 35067539

[13] Lecointre L Dana J Lodi M Akladios C Gallix B. Artificial intelligence-based radiomics models in endometrial cancer: A systematic review. Eur J Surg Oncol (2021) 47:2734–41. doi: 10.1016/j.ejso.2021.06.023 34183201

[14] Aravanis AM Lee M Klausner RD. Next-generation sequencing of circulating tumor DNA for early cancer detection. Cell (2017) 168:571–4. doi: 10.1016/j.cell.2017.01.030 28187279

[15] Phallen J Sausen M Adleff V Leal A Hruban C White J. Direct detection of early-stage cancers using circulating tumor DNA. Sci Transl Med (2017) 9(403):eaan2415. doi: 10.1126/scitranslmed.aan2415 28814544 PMC6714979

[16] Peng Y Mei W Ma K Zeng C. Circulating tumor DNA and minimal residual disease (MRD) in solid tumors: Current horizons and future perspectives. Front Oncol (2021) 11. doi: 10.3389/fonc.2021.763790 PMC8637327 34868984

[17] Tie J Wang Y Cohen J Li L Hong W Christie M. Circulating tumor DNA dynamics and recurrence risk in patients undergoing curative intent resection of colorectal cancer liver metastases: A prospective cohort study. PLoS Med (2021) 18(5):e1003620. doi: 10.1371/journal.pmed.1003620 33939694 PMC8128260

[18] Kim YW Kim YH Song Y Kim HS Sim HW Poojan S. Monitoring circulating tumor DNA by analyzing personalized cancer-specific rearrangements to detect recurrence in gastric cancer. Exp Mol Med (2019) 51(8):93. doi: 10.1038/s12276-019-0292-5 PMC6802636 31395853

[19] Bolivar AM Luthra R Mehrotra M Chen W Barkoh BA Hu P. Targeted next-generation sequencing of endometrial cancer and matched circulating tumor DNA: identification of plasma-based, tumor-associated mutations in early stage patients. Modern Pathol (2019) 32(3):405–14. doi: 10.1038/s41379-018-0158-8 PMC6395490 30315273

[20] Kinde I Bettegowda C Wang Y Wu J Agrawal N Shih IM. Evaluation of DNA from the papanicolaou test to detect ovarian and endometrial cancers. Sci Transl Med (2013) 5(167):167ra4. doi: 10.1126/scitranslmed.3004952 PMC3757513 23303603

[21] Nair N Camacho-Vanegas O Rykunov D Dashkoff M Camacho SC Schumacher CA. Genomic analysis of uterine lavage fluid detects early endometrial cancers and reveals a prevalent landscape of driver mutations in women without histopathologic evidence of cancer: A prospective cross-sectional study. PLoS Med (2016) 13(12):e1002206. doi: 10.1371/journal.pmed.1002206 28027320 PMC5189938

[22] Maritschnegg E Wang Y Pecha N Horvat R van Nieuwenhuysen E Vergote I. Lavage of the uterine cavity for molecular detection of müllerian duct carcinomas: A proof-of-concept study. J Clin Oncol (2015) 33(36):4293–300. doi: 10.1200/JCO.2015.61.3083 PMC4678180 26552420

[23] Li MM Datto M Duncavage EJ Kulkarni S Lindeman NI Roy S. Standards and guidelines for the interpretation and reporting of sequence variants in cancer: A joint consensus recommendation of the association for molecular pathology, American society of clinical oncology, and college of American pathologists. J Mol Diagnostics (2017) 19:4–23. doi: 10.1016/j.jmoldx.2016.10.002 PMC5707196 27993330

[24] Cerami E Gao J Dogrusoz U Gross BE Sumer SO Aksoy BA. The cBio cancer genomics portal: An open platform for exploring multidimensional cancer genomics data. Cancer Discov (2012) 2(5):401–4. doi: 10.1158/2159-8290.CD-12-0095 PMC3956037 22588877

[25] Chakravarty D Gao J Phillips SM Kundra R Zhang H Wang J. OncoKB: A precision oncology knowledge base. JCO Precis Oncol (2017) PO.17.00011. doi: 10.1200/PO.17.00011 PMC5586540 28890946

[26] Tate JG Bamford S Jubb HC Sondka Z Beare DM Bindal N. COSMIC: The catalogue of somatic mutations in cancer. Nucleic Acids Res (2019) 47(D1):D941–7. doi: 10.1093/nar/gky1015 PMC6323903 30371878

[27] Mayakonda A Lin DC Assenov Y Plass C Koeffler HP. Maftools: Efficient and comprehensive analysis of somatic variants in cancer. Genome Res (2018) 28(11):1747–56. doi: 10.1101/gr.239244.118 PMC6211645 30341162

[28] Wang Y Li L Douville C Cohen JD Yen TT Kinde I. Evaluation of liquid from the papanicolaou test and other liquid biopsies for the detection of endometrial and ovarian cancers. Sci Transl Med (2018) 10(433):eaap8793. doi: 10.1126/scitranslmed.aap8793 29563323 PMC6320220

[29] Kyo S Sato S Nakayama K. Cancer-associated mutations in normal human endometrium: Surprise or expected? Cancer Sci (2020) 111(10):3458–67. doi: 10.1111/cas.14571 PMC7541016 32654393

[30] Jiang X Li W Yang J Wang S Cao D Yu M. Identification of somatic mutations in papanicolaou smear DNA and plasma circulating cell-free DNA for detection of endometrial and epithelial ovarian cancers: A pilot study. Front Oncol (2020) 10. doi: 10.3389/fonc.2020.582546 PMC7768029 33381450

[31] Chung YS Woo HY Lee JY Park E Nam EJ Kim S. Mismatch repair status influences response to fertility-sparing treatment of endometrial cancer. Am J Obstetrics Gynecol (2021) 224(4):370.e1–370.e13. doi: 10.1016/j.ajog.2020.10.003 33039397

[32] Kurose K Gilley K Matsumoto S Watson PH Zhou XP Eng C. Frequent somatic mutations in PTEN and TP53 are mutually exclusive in the stroma of breast carcinomas. Nat Genet (2002) 32(3):355–7. doi: 10.1038/ng1013 12379854

[33] Janiec-Jankowska A Konopka B Goluda C Najmoaa U. TP53 mutations in endometrial cancers relation to PTEN gene defects. Int J Gynecol Cancer (2010) 20(2):196–202. doi: 10.1111/IGC.0b013e3181c83675 20169661

[34] Bosse T ter Haar NT Seeber LM Diest PJV Hes FJ Vasen HF. Loss of ARID1A expression and its relationship with PI3K-akt pathway alterations, TP53 and microsatellite instability in endometrial cancer. Modern Pathol (2013) 26(11):1525–35. doi: 10.1038/modpathol.2013.96 23702729

[35] Chandler RL Damrauer JS Raab JR Schisler JC Wilkerson MD Didion JP. Coexistent ARID1A-PIK3CA mutations promote ovarian clear-cell tumorigenesis through pro-tumorigenic inflammatory cytokine signalling. Nat Commun (2015) 6:6118. doi: 10.1038/ncomms7118 25625625 PMC4308813

