# Supplementary figures and images for: Feasibility and clinical applicability of genomic profiling based on cervical smear samples in patients with endometrial cancer
Source: Front Oncol. 2022 Aug 5;12:942735. doi: 10.3389/fonc.2022.942735 (PMC9389008; doi:10.3389/fonc.2022.942735)

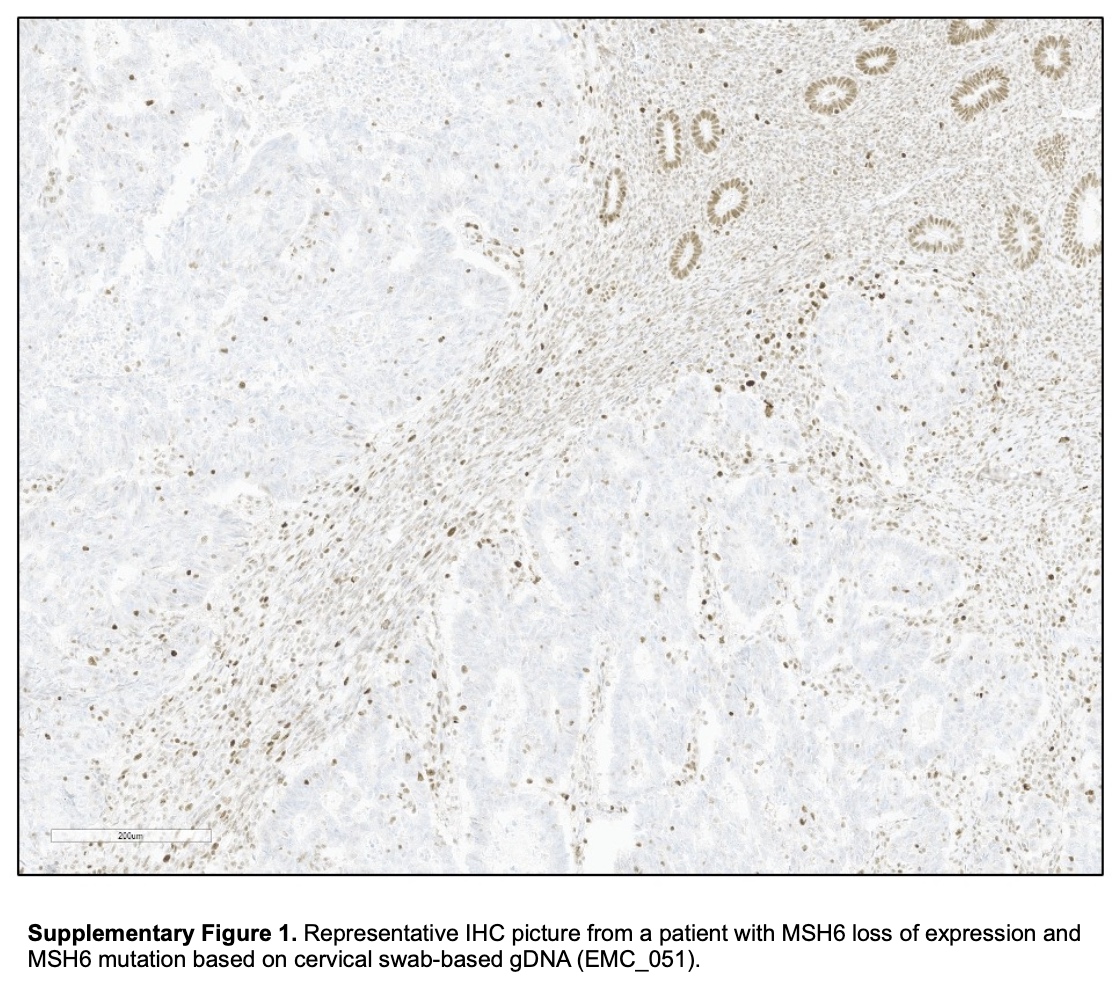

Supplement: Supplementary Figure 1 — Representative IHC picture from a patient with MSH6 loss of expression and MSH6 mutation based on cervical swab-based gDNA (EMC_051). [file Image_1.jpeg]

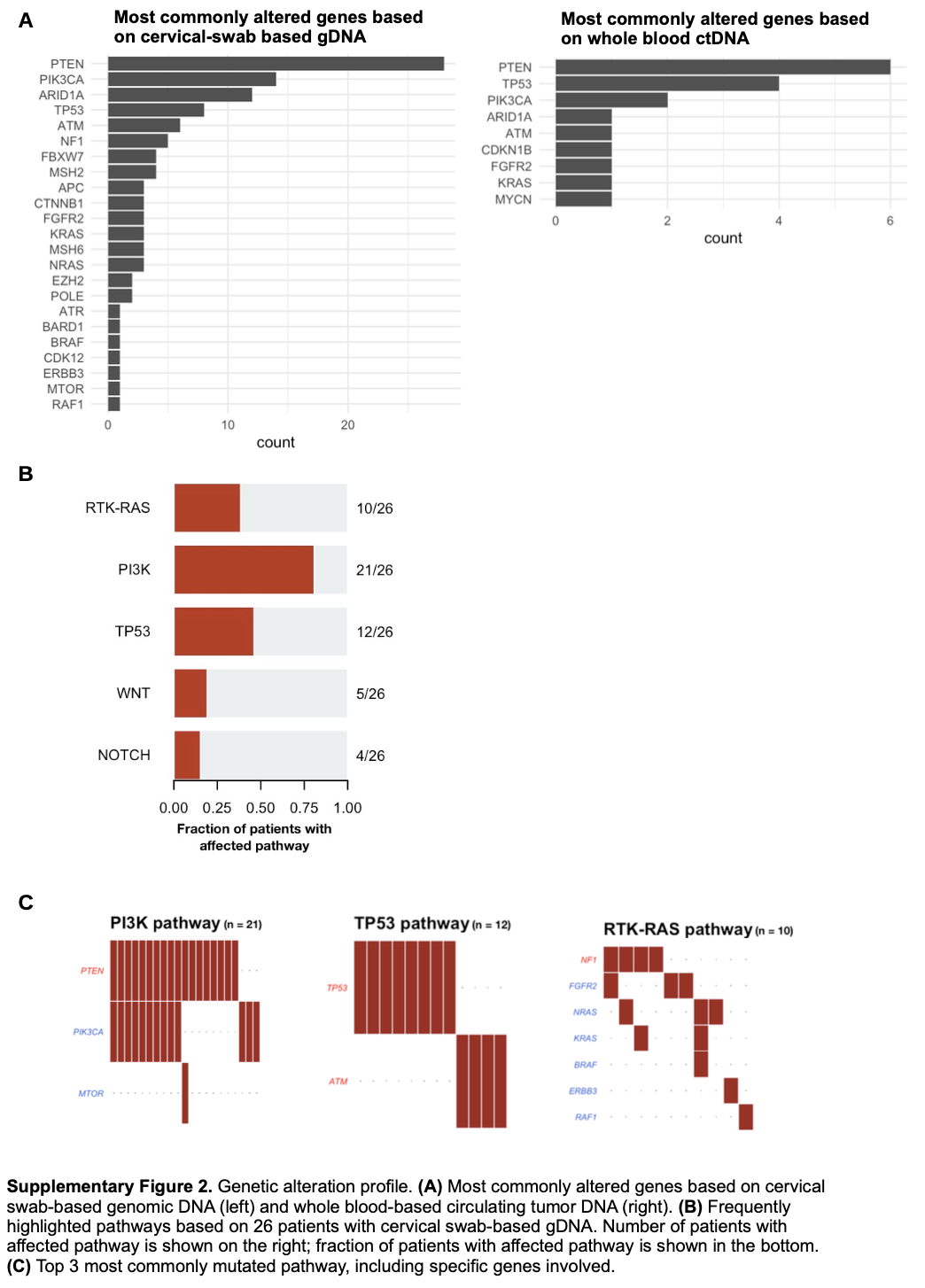

Supplement: Supplementary Figure 2 — Genetic alteration profile. (A) Most commonly altered genes based on cervical swab-based genomic DNA (left) and whole blood-based circulating tumor DNA (right). (B) Frequently highlighted pathways based on cervical swab-based gDNA from 26 patients. Number of patients with affected pathway is shown on the right; fraction of patients with affected pathway is shown on the bottom. (C) Top 3 most frequently mutated pathway, including specific genes involved. [file Image_2.jpeg]

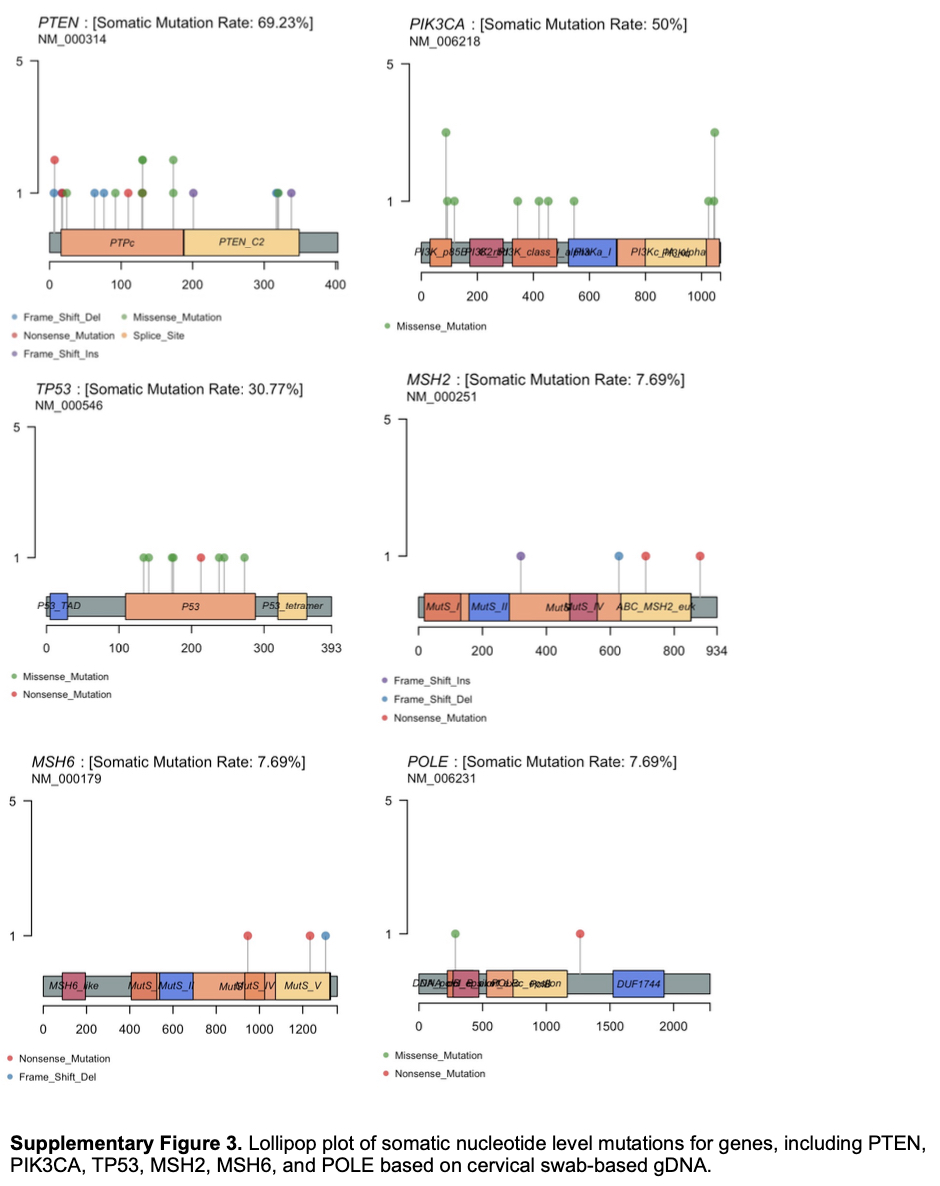

Supplement: Supplementary Figure 3 — Lollipop plot of somatic nucleotide level mutations for genes, including PTEN, PIK3CA, TP53, MSH2, MSH6, and POLE based on cervical swab-based gDNA. [file Image_3.jpeg]
