# Supplementary material for: Feasibility and clinical applicability of genomic profiling based on cervical smear samples in patients with endometrial cancer
Source: Front Oncol. 2022 Aug 5;12:942735. doi: 10.3389/fonc.2022.942735 (PMC9389008; doi:10.3389/fonc.2022.942735)
Supplement: Supplementary file 4 [file Table_1.docx]

**Supplementary Table 1.** Performance of cervical swab-based genomic DNA (gDNA) for predicting endometrial cancer versus benign uterine disease in a sub cohort (excluding 20 stage IA patients).

|  | | Reference | |
| --- | --- | --- | --- |
|  |  | No cancer | Cancer |
| Prediction by cervical swab-based gDNA | Negative | 11 | 2 |
|  | Positive | 0 | 17 |
| Accuracy: 0.93 (95% CI 0.78 – 0.99) | | | |
| Sensitivity: 0.89 | | | |
| Specificity: 1.00 | | | |
| Positive predictive value: 1.00 | | | |
| Negative predictive value: 0.85 | | | |

**Supplementary Table 2.** Detailed clinical characteristics of endometrial cancer patients.

| Cervical swab | Matched blood | Age | Stage | Histology | Grade | Size (cm) | Depth (cm) | Lymph node involvement | Detection by cervical swab-based gDNA | Detection by whole blood-based ctDNA | Detection by conventional Pap smear |
| --- | --- | --- | --- | --- | --- | --- | --- | --- | --- | --- | --- |
| EMC_004 | EMC_005 | 76 | IA | endometrioid | 3 | 1.8 | 0.3/1.5 | No | none | none | negative |
| EMC_035 | EMC_034 | 60 | IA | endometrioid | 1 | 1.5 | 0.4/1.4 | No | none | none | negative |
| EMC_041 | EMC_040 | 62 | IV | carcinosarcoma | other | 3.8 | 1.3/1.4 | Yes | none | detected | negative |
| EMC_045 | EMC_043 | 39 | IA | endometrioid | 1 | 0 | 0 | No | none | none | negative |
| EMC_051 | EMC_050 | 45 | IA | endometrioid | 1 | 0.5 | 0 | No | detected | detected | negative |
| EMC_053 |  | 55 | IA | endometrioid | 1 | 0 | 0 | No | none | not done | negative |
| EMC_060 |  | 44 | IA | endometrioid | 2 | 2.5 | 0.23/0.35 | No | detected | not done | negative |
| EMC_068 | EMC_065 | 73 | IV | endometrioid | 2 | 2.5 | 0.1/1.4 | No | detected | none | negative |
| EMC_078 | EMC_077 | 61 | IB | endometrioid | 1 | 2.4 | 1.5/2.2 | No | detected | none | negative |
| EMC_102 |  | 47 | IA | leiomyosarcoma | other | 0 | 0 | No | none | not done | negative |
| EMC_008 | EMC_009 | 78 | IB | endometrioid | 3 | 7.2 | 0.8/0.8 | No | detected | detected | negative |
| EMC_013 | EMC_010 | 61 | II | endometrioid | 2 | 3.2 | 0.2/1.1 | No | detected | none | carcinoma |
| EMC_015 | EMC_012 | 66 | IA | endometrioid | 2 | 2.5 | 0.3/1.2 | No | detected | none | negative |
| EMC_017 | EMC_016 | 53 | IA | endometrioid | 2 | 4.3 | 0.7/1.5 | No | detected | none | carcinoma |
| EMC_030 | EMC_029 | 72 | IB | endometrioid | 2 | 3.3 | 0.9/1.5 | No | detected | none | negative |
| EMC_039 | EMC_038 | 64 | IA | endometrioid | 2 | 2 | 0.4/0.9 | No | detected | none | negative |
| EMC_055 | EMC_054 | 55 | III | endometrioid | 2 | 2.5 | 1.5/2.2 | Yes | detected | none | not_done |
| EMC_057 | EMC_056 | 63 | IB | endometrioid | 2 | 2.5 | 0.8/1.3 | No | detected | none | carcinoma |
| EMC_070 | EMC_069 | 49 | IA | endometrioid | 2 | 1.1 | 0.4/1.5 | No | detected | none | negative |
| EMC_072 | EMC_071 | 45 | IB | endometrioid | 2 | 3 | 1.0/1.4 | No | detected | none | carcinoma |
| EMC_096 | EMC_095 | 49 | IA | endometrioid | 1 | 3.5 | 0 | No | detected | none | negative |
| EMC_092 |  | 56 | IA | endometrioid | 1 | 1.6 | 0 | No | none | not done | negative |
| EMC_099 | EMC_098 | 40 | IV | endometrioid | 3 | 4.5 | 3.2/3.2 | Yes | detected | detected | negative |
| EMC_101 | EMC_100 | 44 | III | endometrioid | 2 | 7 | 0.9/1.4 | Yes | detected | detected | negative |
| EMC_115 | EMC_114 | 43 | III | endometrioid | 2 | 5.2 | 1.8/2.3 | Yes | detected | detected | detected |
| EMC_117 | EMC_116 | 52 | IB | endometrioid | 2 | 2.5 | 1.1/2 | No | detected | none | detected |
| EMC_119 | EMC_118 | 77 | III | serous | 3 | 5.2 | 1.4/1.4 | No | detected | none | detected |
| EMC_126 |  | 52 | IA | endometrioid | 1 | 1.5 | 0.2/1.9 | No | none | not done | none |
| EMC_129 | EMC_128 | 57 | IA | carcinosarcoma | other | 7 | 0.3/1.5 | No | detected | none | detected |
| EMC_136 | EMC_137 | 48 | IA | endometrioid | 2 | 5.3 | 0.3/1.6 | No | detected | none | detected |
| EMC_138 |  | 53 | IA | endometrioid | 1 | 0.5 | 0 | No | none | not done | none |
| EMC_143 | EMC_144 | 61 | IA | endometrioid | 3 | 0.2 | 0 | No | none | detected | none |
| EMC_147 | EMC_148 | 78 | IB | endometrioid | 2 | 3 | 1.8/2 | No | detected | none | detected |
| EMC_150 | EMC_153 | 54 | III | endometrioid | 2 | 5 | 1.9/2.3 | Yes | detected | detected | detected |
| EMC_154 | EMC_155 | 56 | III | stromalsarcoma | 3 | 14 | 3.5/3.5 | No | detected | detected | detected |
| EMC_159 |  | 60 | IA | endometrioid | 1 | 0.5 | 0 | No | none | not done | none |
| EMC_166 | EMC_167 | 55 | III | endometrioid | 2 | 2.5 | 1.8/2.0 | Yes | none | none | none |
| EMC_168 |  | 65 | IV | endometrioid | 2 | 6 | 2.1/2.2 | No | detected | not done | detected |
| EMC_170 | EMC_171 | 77 | IA | endometrioid | 2 | 1.8 | 0.5/1.6 | No | none | none | none |

*gDNA* genomic DNA, *ctDNA* circulating tumor DNA
